# Repurposing ebselen for decolonization of vancomycin-resistant enterococci (VRE)

**DOI:** 10.1371/journal.pone.0199710

**Published:** 2018-06-28

**Authors:** Ahmed AbdelKhalek, Nader S. Abutaleb, Haroon Mohammad, Mohamed N. Seleem

**Affiliations:** 1 Department of Comparative Pathobiology, College of Veterinary Medicine, Purdue University, West Lafayette, Indiana, United States of America; 2 Purdue Institute of Inflammation, Immunology, and Infectious Disease, West Lafayette, Indiana, United States of America; Cornell University, UNITED STATES

## Abstract

Enterococci represent one of the microbial world’s most challenging enigmas. Colonization of the gastrointestinal tract (GIT) of high-risk/immunocompromised patients by enterococci exhibiting resistance to vancomycin (VRE) can lead to life-threating infections, including bloodstream infections and endocarditis. Decolonization of VRE from the GIT of high-risk patients represents an alternative method to suppress the risk of the infection. It could be considered as a preventative measure to protect against VRE infections in high-risk individuals. Though multiple agents (ramoplanin and bacitracin) have been evaluated clinically, no drugs are currently approved for use in VRE decolonization of the GIT. The present study evaluates ebselen, a clinical molecule, for use as a decolonizing agent against VRE. When evaluated against a broad array of enterococcal isolates *in vitro*, ebselen was found to be as potent as linezolid (minimum inhibitory concentration against 90% of clinical isolates tested was 2 μg/ml). Though VRE has a remarkable ability to develop resistance to antibacterial agents, no resistance to ebselen emerged after a clinical isolate of vancomycin-resistant *E*. *faecium* was serially-passaged with ebselen for 14 days. Against VRE biofilm, a virulence factor that enables the bacteria to colonize the gut, ebselen demonstrated the ability to both inhibit biofilm formation and disrupt mature biofilm. Furthermore, in a murine VRE colonization reduction model, ebselen proved as effective as ramoplanin in reducing the bacterial shedding and burden of VRE present in the fecal content (by > 99.99%), cecum, and ileum of mice. Based on the promising results obtained, ebselen warrants further investigation as a novel decolonizing agent to quell VRE infection.

## Introduction

Enterococcal infections represent one of the major challenges facing healthcare providers worldwide, in part because of the uncanny ability of enterococci to acquire or develop resistance to antibiotics. In addition to their intrinsic resistance to many antibiotics, enterococci have developed resistance to many antibiotics either through mutations in the target gene/protein of an antibiotic or through acquisition of foreign genetic material, this is particularly seen in species of *Enterococcus faecium* and *Enterococcus faecalis* [1–3]. The emergence of clinical isolates exhibiting resistance to vancomycin (termed vancomycin-resistant enterococci or VRE) has been troubling as these isolates are often co-resistant to other classes of antibiotics including β-lactams [2]. Though newer antibiotics such as linezolid, daptomycin, tigecycline and quinupristin/dalfopristin remain effective treatment options clinically, several cases of enterococcal resistance against all the aforementioned drugs have been reported [4–9]. As a result, infections caused by drug-resistant enterococci are one of the major and ascending challenges present in healthcare settings [10]. Recently, this was affirmed when the World Health Organization listed vancomycin-resistant *E*. *faecium* as one of the high priority pathogens for research and development of new antibiotics and novel strategies to combat infections [11]. One novel strategy that warrants further investigation is identifying agents capable of decolonizing VRE from the gastrointestinal tract (GIT) of high-risk patients susceptible to infection [12].

Both *E*. *faecium* and *E*. *faecalis* are normal inhabitants of the human GIT, and their count remains constant due to a natural trait of the GIT know as colonization resistance [13]. Colonization resistance is the active ability of the host to eliminate pathogens from the GIT. The most important element that enhances colonization resistance is the presence of healthy gut microbiota. Administration of broad-spectrum antibiotics is a common practice for patients undergoing solid organ transplants and immunocompromised patients at high-risk of bacterial infection. However, these antibiotics are capable of disrupting the integrity of the normal GI bacterial consortium allowing for colonization by antibiotic-resistant enterococci [10]. An established enterococcal colonization can persist for months to years during which the carrier serves as a springboard for infection. Enterococcal colonization of the intestinal mucosal surface has been identified as a key initial step that permitted bacterial invasion of the bloodstream [10,14,15]. Given the significance of gut colonization in the development of enterococcal infections, it is quite surprising that no drug is currently approved for the decolonization of multidrug-resistant *Enterococcus* [12,15]. Several antibiotics (including bacitracin) have been investigated for use as decolonizing agents against enterococci but all suffered from poor patient tolerability or limited efficacy [16,17]. Another molecule, ramoplanin has been investigated in clinical trials to decolonize VRE from the GIT of susceptible patients. Though this molecule did successfully reduce the burden of VRE in the GIT, patients suffered a high rate of recurrence after treatment was stopped; furthermore ramoplanin had a negative impact on the microbiota as it promoted overgrowth of Gram-negative pathogens [17,18]. Thus, there represents an unaddressed need to find new, safe molecules and drugs that can be used as decolonizing agents against VRE.

One approach for discovering novel decolonizing agents is via drug repurposing. This approach significantly decreases the high innovation cost and time normally associated with bringing a new drug to the clinic [19]. Using this approach, we recently discovered ebselen, a multifunctional organoselenium molecule in clinical trials, it possesses a potent antibacterial activity against important Gram-positive bacterial pathogens (including VRE) [20,21]. Ebselen is being evaluated for various applications including cancer, cardiovascular disorders and kidney disorders [22,23]. The activity of ebselen was established against a wide range of microbes including several *staphylococcus* strains, *Escherichia coli*, *Bacillus subtilis*, *Helicobacter pylori*, *Candida albicans* and *Aspergillus niger* [24]. Despite its known antimicrobial activity, the potential to use ebselen as a decolonizing agent against VRE has not been investigated. Thus, the present study evaluates the activity of ebselen against a wider panel of enterococcal clinical isolates, investigates the ability of VRE to develop resistance to ebselen, examines the efficacy of ebselen to disrupt VRE biofilm, and evaluates ebselen’s ability to decolonize VRE from the GIT using a murine VRE colonization reduction model.

## Results

### Activity of ebselen against enterococcal isolates *in vitro*

The antibacterial activity of ebselen, linezolid, vancomycin, and ramoplanin was evaluated against 27 strains of enterococci from humans and animals. Most of the tested strains, Table 1, were resistant to vancomycin. Utilizing the broth microdilution assay, ebselen was found to possess potent antibacterial activity against all the tested isolates (Table 1). Against both vancomycin-resistant and vancomycin-sensitive strains, ebselen inhibited growth of 50% of all the tested isolates (MIC_50_) at a concentration of 1 μg/mL. Against 90% of the isolates tested (MIC_90_), ebselen’s inhibited growth at 2 μg/mL. The MIC_50_ and MIC_90_ for linezolid were equal to ebselen. The MIC_50_ and MIC_90_ for ramoplanin was two-fold higher than the values obtained for ebselen. Vancomycin’s MIC exceeded 128 μg/mL against more than 90% of the clinical isolates tested.

**Table 1:** The minimum inhibitory concentration (MIC, μg/mL) of ebselen and control antibiotics against enterococci clinical isolates.

Strains	MIC (μg/mL)				Source
	Ebselen	Linezolid	Ramoplanin	Vancomycin	
*E*. *faecium*, UAA714	0.5	1	2	>128	Aix-en-Provence, France.
*E*. *faecium*, HF50106	1	1	0.25	128	Swine feces, Michigan, USA.
*E*. *faecalis*, TX0104	0.5	1	2	>128	Blood of a patient with endocarditis, Connecticut, USA.
*E*. *faecalis*, S613	1	1	2	>128	Blood of a 64-year-old female hemodialysis patient with fatal bacteremia
*E*. *faecium*, E1578	0.5	2	2	4	Feces of a miniature pig in Germany.
*E*. *faecium*, UAA945	≤ 0.25	0.5	2	>128	New York, USA.
*E*. *faecium*, ERV165	1	1	0.5	>128	Feces collected in Colombia.
*E*. *faecium*, Patient #2–1	0.5	1	4	>128	Stool of a human patient prior to bacteremia.
*E*. *faecium*, E0269	0.5	1	1	128	Turkey feces in the Netherlands.
*E*. *faecium*, HF50104	1	1	0.5	>128	Swine feces, Michigan, USA.
*E*. *faecium*, 503	1	1	2	>128	Human isolate from the United States.
*E*. *faecium*, E417	1	1	1	>128	Human blood, Ecuador.
*E*. *faecalis*, R712	1	1	2	>128	Blood of a 64-year-old female hemodialysis patient with fatal bacteremia.
*E*. *faecium*, ERV102	2	1	1	>128	Human oral sputum, Colombia.
*E*. *faecium*, Patient #1–1	1	16	2	>128	VRE isolated from the stool of a human patient prior to bacteremia.
*E*. *faecium*, E0164	2	1	1	>128	Turkey feces, Netherlands.
*E*. *faecium*, HF50105	1	1	0.25	>128	Swine feces, Michigan, USA.
*E*. *faecium*, E1620	1	1	2	2	Human blood, Netherlands.
*E*. *faecium*, E2620	1	1	2	2	Human Blood, Netherlands.
*E*. *faecalis*, SF24413	1	1	2	64	Urine, Michigan, USA.
*E*. *faecium*, E0120	1	1	2	>128	Ascites fluid, Netherlands.
*E*. *faecalis*, SF28073	1	1	4	>128	VRE isolated in 2003 from a human urine sample obtained in Michigan, USA.
*E*. *faecium*, E1071	0.5	1	2	>128	Netherlands, hospital surveillance program.
*E*. *faecium*, Patient #3–1	1	1	2	>128	Stool of a human patient.
*E*. *faecalis*, ERV103	0.5	1	4	>128	Human, Bogota, Colombia.
*E*. *faecium*, E1604	4	2	2	≤1	Cheese, Norway.
*E*. *faecalis*, B3336	2	0.5	4	≤1	Human blood, United States.
MIC50	1	1	2	>128	
MIC90	2	2	4	>128	

### Time-kill kinetics of ebselen against VRE

**Fig 1:**
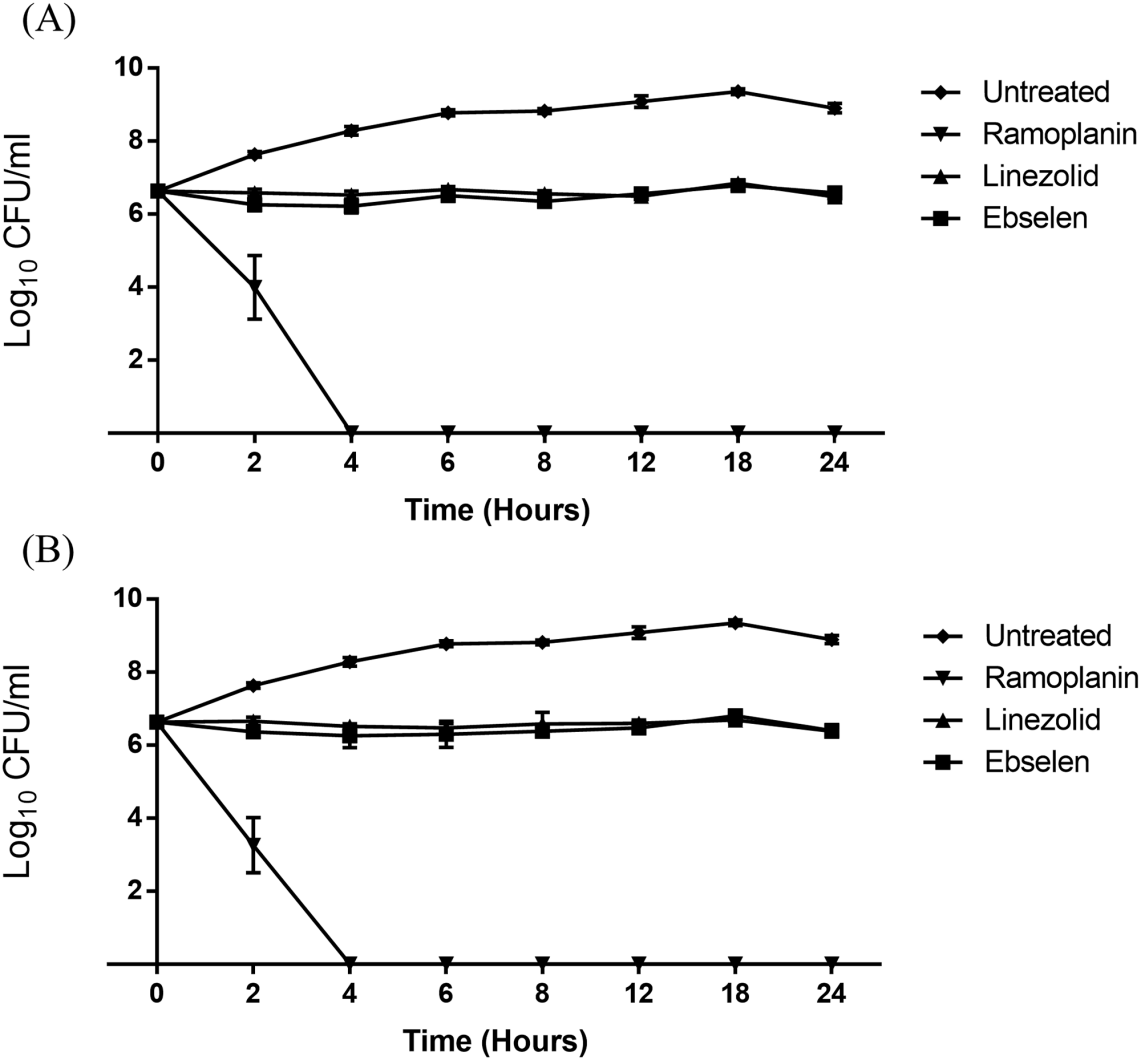
Time-kill assay for ebselen and the control antibiotics, linezolid and ramoplanin. Tested at **(A)** 3 × MIC and **(B)** 6 × MIC. *E*. *faecium* HM-952 was aerobically incubated with the indicated concentrations of the drugs, in triplicate, for 24 hours at 37 °C and samples were collected at the indicated time points to enumerate bacteria.

After confirming the potent antibacterial activity of ebselen, we sought to investigate if ebselen is bacteriostatic or bactericidal against VRE. Using a standard time-kill assay, ebselen was found to exhibit a bacteriostatic mode of action at two different concentrations (3×MIC and 6×MIC, against vancomycin-resistant *E*. *faecium*) (Fig 1). Linezolid exhibited a similar pattern of activity to ebselen at both concentrations. In contrast, ramoplanin was found to exhibit rapid bactericidal action, completely reducing the burden of VRE to zero after four hours (at both test concentrations). A drug was only referred to as bactericidal if it could inhibit ≥ 99.9% of the bacterial burden.

### Evaluation of resistance development to ebselen

**Fig 2:**
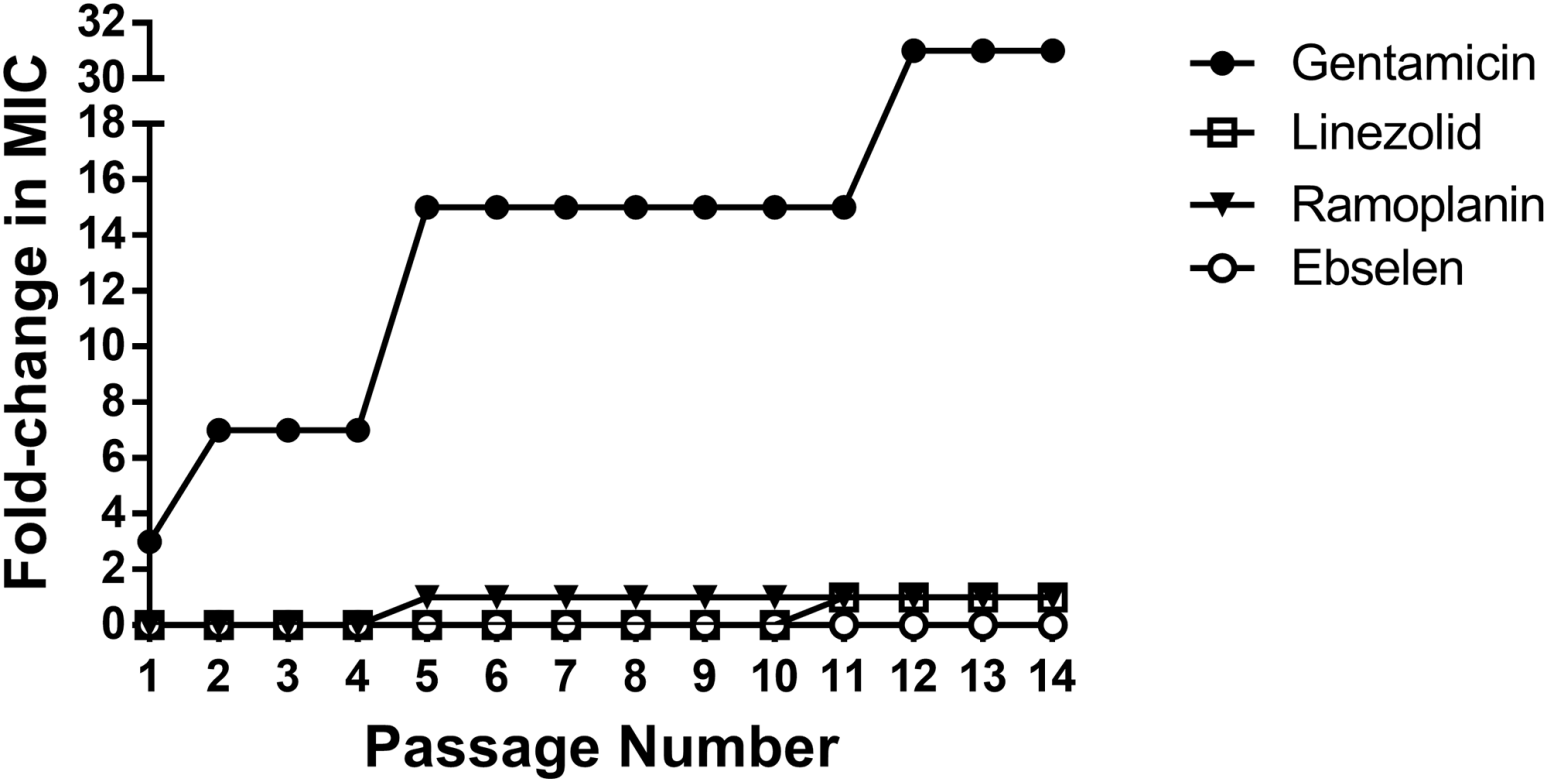
Multi-step resistance selection of *E*. *faecium* HM-952 in presence of ebselen, gentamicin, linezolid, or ramoplanin. The MIC of all the test agents was determined daily (for 14 passages) to test for the development of resistance (increase in MIC) to the tested isolate. A 4-fold increase in the MIC is indicative of resistance formation.

Recognizing the great propensity of enterococci to develop resistance to antibacterial agents [25], we were curious to test whether or not VRE can develop resistance to ebselen. To investigate this point, ebselen was evaluated via a multi-step resistance selection experiment against vancomycin-resistant *E*. *faecium*. As depicted in Fig 2, *E*. *faecium* remained sensitive to ebselen even after 14 consecutive passages (no change in the MIC was observed). Similar effects were observed with linezolid and ramoplanin (only one-fold increase in MIC). In contrast, resistance to gentamicin emerged rapidly. After the second passage, the MIC of gentamicin increased seven-fold. The MIC continued to increase, resulting in a 31-fold change in the MIC of gentamicin at the end of the 14 passages. Although there was 1-fold increase in the MICs of Linezolid and ramoplanin, unlike ebselen, they did not cross the 4-fold cutoff limit that distinguishes sensitivity from resistance [26].

### Antibiofilm activity of ebselen

**Fig 3:**
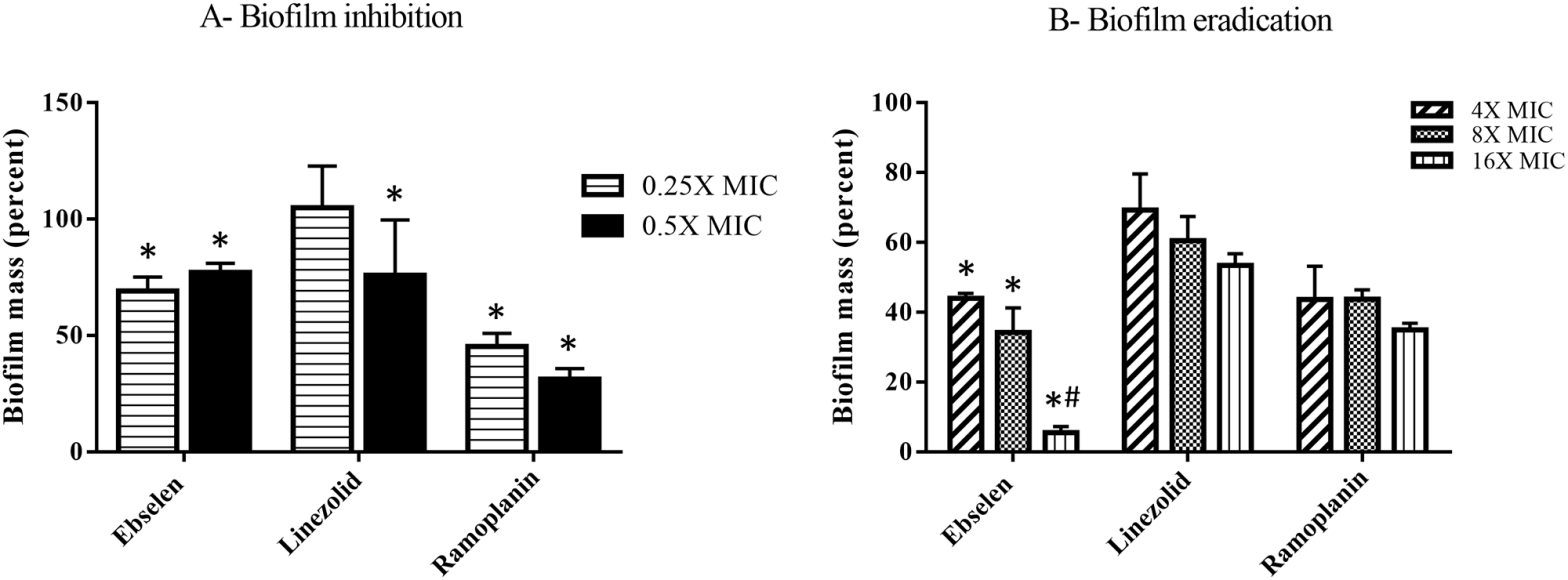
The anti-biofilm activity of ebselen against *E*. *faecalis* NR-31972. **(A) Biofilm inhibition activity of ebselen**. Sub-inhibitory concentrations of each drug were added to bacteria in tryptic soya broth (TSB) + 1% glucose and incubated for 24 hours at 37 °C. The biofilm mass (OD_595_) was measured after staining with crystal violet and destaining with ethanol. Data is presented as biofilm mass relative to DMSO-treated wells, (*) indicates significant difference from DMSO treated wells using 2-way ANOVA with Dunnett’s pairwise comparison (P< 0.001). **(B) Biofilm eradication activity of ebselen**. Bacteria were incubated for 24 hours in TSB + 1% glucose to allow for the formation of mature biofilm. Supra-inhibitory concentrations of the drugs were then added and incubated with the bacterial biofilm for additional 24 hours before the biofilm density was measured (OD_595_) by crystal violet staining. Data is presented as biofilm mass relative to DMSO-treated wells, (*) indicates significant difference from linezolid-treated wells, while (^#^) indicates significant difference from ramoplanin-treated wells using 2-way ANOVA with Dunnett’s post hoc comparison test at P<0.05.

We next moved to investigate if ebselen would be capable of interfering with a key virulence factor, biofilm formation, important for GIT colonization by VRE. *E*. *faecalis* NR-31972 was used given it forms strong, mature biofilm in microtiter plates. Interestingly, ebselen was found to exhibit a concentration-dependent inhibition of VRE biofilm formation. Ebselen was found to inhibit about 30% of VRE biofilm formation at 0.25×MIC and 0.5×MIC (Fig 3A). Linezolid, in contrast, was only effective at inhibiting biofilm formation at 0.5×MIC. Ramoplanin inhibited biofilm formation by 55% (at 0.25×MIC) and 70% (at 0.5×MIC).

We also investigated the ability of ebselen to disrupt mature, adherent VRE biofilm. Ebselen was superior to all other tested drugs in eradicating established VRE biofilm. In a concentration-dependent manner, ebselen reduced mature VRE biofilm by 55% (at 4×MIC), 65% (at 8×MIC), and 95% (at 16×MIC), respectively (Fig 3B). At the same three concentrations, linezolid reduced biofilm mass by 30% (4×MIC), 40% (8×MIC), and 45% (16×MIC) while ramoplanin reduced the biofilm mass by 55% (4×MIC), 55% (at 8×MIC), and 65% (16×MIC). (*) indicates significant difference from linezolid-treated wells, while (^#^) indicates significant difference from ramoplanin-treated wells using 2-way ANOVA with Dunnett’s post hoc comparison test at P<0.001.

### *In vivo* assessment of ebselen in a VRE colonization reduction mice model

**Fig 4:**
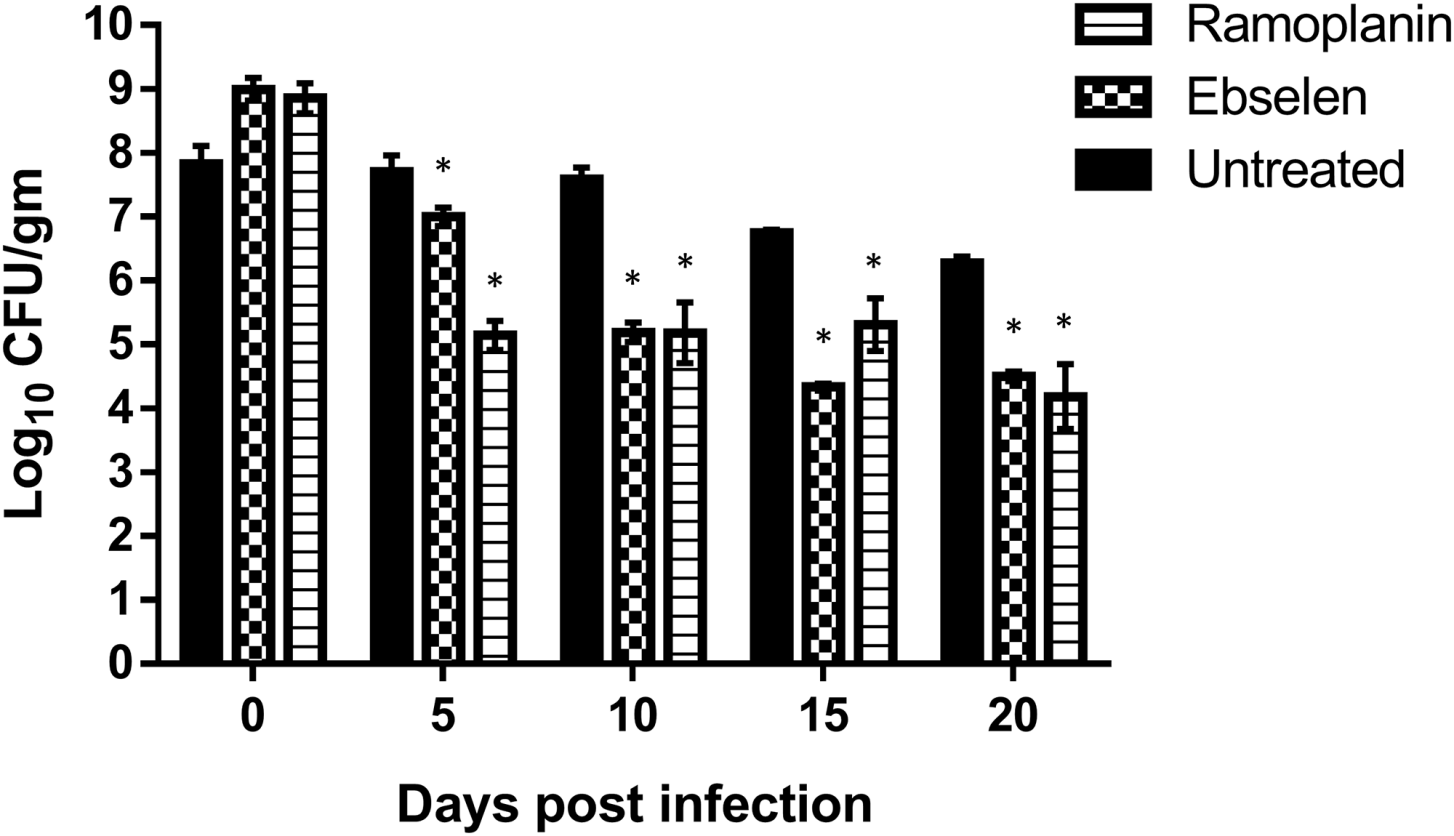
Bacterial counts of *E*. *faecium* HM-952 in the fecal samples of infected mice. Infected mice were orally treated with ebselen (0.5 mg/kg) and ramoplanin (10 mg/kg) daily for 8 days. One group was left untreated. Fecal samples were freshly collected from mice in each group on days 0, 5, 10, 15, and 20 post treatment. (*) denotes significant difference from the untreated group (P < 0.001).

After confirming the potent *in vitro* effect of ebselen against both planktonic VRE and VRE biofilm, we moved to confirm ebselen’s ability to decolonize VRE from the GIT of infected mice. Guided by the protocol of Ubeda *et al*. [15], ebselen was used to treat mice colonized with VRE. The effects of ebselen (10 mg/kg) and ramoplanin (10 mg/kg) were evaluated based on their ability to decrease bacterial burden in the stool samples of infected mice. Both ebselen (0.8-log_10_ reduction in CFU/mL) and ramoplanin (2.5-log_10_ reduction in CFU/mL) significantly reduced the burden of VRE in the stool, relative to mice in the untreated group, starting at day five (Fig 4A). Ebselen continued to reduce the burden of VRE by 2.4-log_10_ reduction by day 15 and additional 1.7-log_10_ reduction by day 20. This was similar to the result obtained for ramoplanin which reduced the burden of VRE (relative to untreated mice) in fecal samples by 2.5-log_10_ on day 5, 2.4-log_10_ reduction (day 10), 1.4-log_10_ reduction (day 15), and 2.1-log_10_ reduction by day 20.

**Fig 5:**
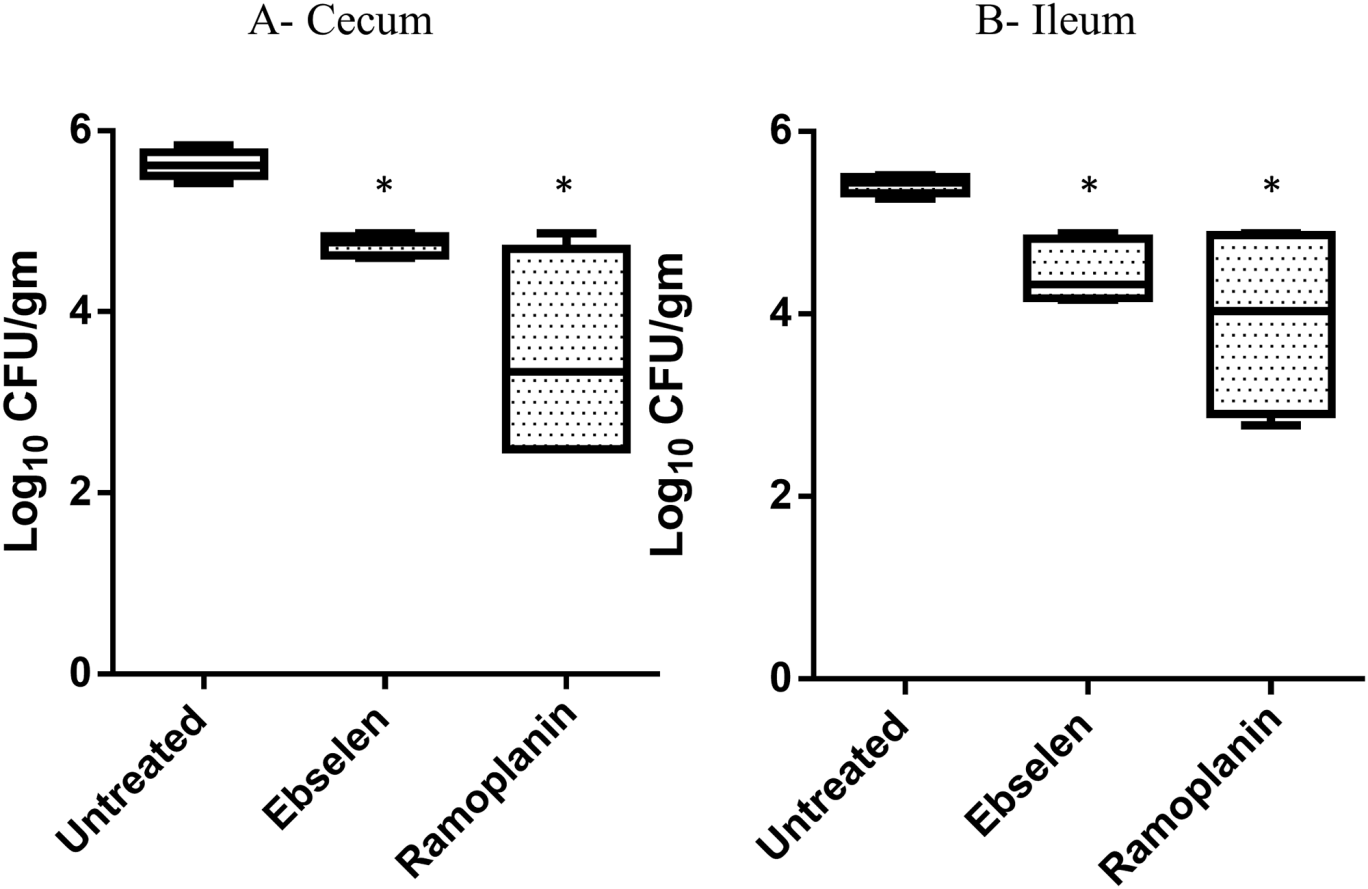
Bacterial counts of *E*. *faecium* HM-952 in (A) cecum and (B) ileum contents of mice. Infected mice (n = 5 per group) were orally treated with ebselen (0.5 mg/kg) or ramoplanin (10 mg/kg) daily for eight days. One group was left untreated. Cecum and ileum contents were collected one day after the last treatment was administered (day 21 of experiment). Asterisk (*) denotes significant difference from the untreated group (P < 0.05). No significant difference was found between ebselen-treated and ramoplanin-treated groups.

In addition to examining the presence of VRE in stool samples of infected mice, the burden of VRE present in the cecum and ileum of mice was determined. One day after the final treatment was administered, mice were humanely euthanized and the cecum and ileum were aseptically removed and homogenized to determine viable bacterial CFU. Similar to results obtained from the fecal samples, ebselen and ramoplanin significantly diminished the burden of VRE in both the cecal and ileal contents (Fig 5). Ebselen reduced the burden of VRE by 0.9-log_10_ in the ceca and generated a one-log_10_ reduction in the ilea of mice. Ramoplanin generated a 2.1-log_10_ reduction of VRE in the ceca and 1.5-log_10_ reduction in the ilea of infected mice.

## Discussion

The challenge of multidrug-resistant enterococcal infection continues to pose a threat to patients in healthcare facilities. Enterococcal infections represent about 9% of all the healthcare-associated infections in the United States of America alone. Due to their broad tissue tropism, enterococci can infect a wide variety of human organs. Enterococci, principally *E*. *faecium* and *E*. *faecalis*, are the causative agent of about 15% of bloodstream infections, urinary tract infections and surgical site infections. Moreover, bloodstream infections can advance to cause infective endocarditis that can be fatal in up to 46% of the cases [10,27]. Treatment of enterococcal infections has become increasingly challenging given the remarkable ability of enterococci to develop resistance to antibacterial agents [3,25]. The emergence of clinical isolates of *E*. *faecium* and *E*. *faecalis* exhibiting resistance to vancomycin presents a formidable challenge given these strains are often co-resistant to other antibiotics. Though newer antibiotics such as linezolid have become the mainstays of treatment for VRE infections, these antibiotics are not immune to resistance development. As highlighted in a recent study by Bi *et al*, enterococci exhibiting resistance to linezolid represents an emerging problem globally [28]. Interestingly, the ability of enterococci to develop resistance against antibiotics is more prominent in strains of *E*. *faecium* moreso than *E*. *faecalis*. About 79% percent of *E*. *faecium* infections are vancomycin-resistant while only nine percent of *E*. *faecalis* infections are vancomycin-resistant [29]. Given the potential challenge of treating VRE infections once they arise, alternative approached to combating infection are needed. One such strategy is decolonizing the GIT of patients susceptible to infection by VRE.

One of the leading events that heralds enterococcal infections is gastrointestinal colonization. Enterococci normally reside in the human GIT as a member of the gut microflora. In normal settings, the population of enterococci remains in balance with the other members of the healthy bacterial consortium of the gut. However, administration of broad-spectrum antibiotics can disrupt the integrity of the normal gut flora leading to diminished ability to resist enterococcal overgrowth including strains of antibiotic-resistant enterococci. This GIT colonization has two major consequences: infection of the colonized individual and cross-transmission of enterococci to other patients residing within the same healthcare facility [15,29–31]. Decolonization is not typically performed for patients with VRE because the current decolonization strategies, including antibiotics, bowel washing and administration of probiotics, suffer poor tolerability and/or limited efficacy [16,17,32]. Important qualities to seek in a decolonizing agent for VRE include potent antibacterial activity against VRE, stability to resistance development, safety to humans, and efficacy to decrease the burden of VRE in the intestinal tract. To date, no agent exists that possesses all of these qualities. Thus there remains a need to identify and develop new decolonizing agents effective against VRE.

Ebselen is an organoselenium compound that is being investigated for the treatment of various conditions and has been proven to be safe for human use [33,34]. Ebselen is/has been evaluated for its preventive and treatment activates against several diseases such as cancer, ischemic stroke, hearing loss, diabetes-related atherosclerosis and nephropathy and bipolar disorder [22,23,35–37]. More recently, ebselen was found to possess potent antibacterial activity against both VRE and methicillin-resistant *Staphylococcus aureus* (MRSA). While the activity of ebselen against MRSA has been intensively studied [20,21,38,39], ebselen’s activity against VRE still needs further evaluation. Herein, the capability of ebselen to serve as a novel decolonizing agent against VRE was investigated.

Initially, the antibacterial activity of ebselen was evaluated against more than 20 clinical isolates of VRE. Ebselen inhibited growth of the vast majority of these isolates at concentrations as low as 2 μg/mL, similar to linezolid. It is important to highlight that ebselen was effective against both *E*. *faecalis* and *E*. *faecium*, unlike some of the anti-enterococcal agents that have been used previously (such as quinupristin-dalfopristin) [4,25]. Likewise, ebselen was active against both VRE and vancomycin-sensitive strains. When evaluated against VRE in a time-kill assay, ebselen was found to exert a bacteriostatic activity, similar to linezolid.

Given enterococci’s remarkable ability to develop resistance against antibacterial agents, we were compelled to test whether ebselen-resistant mutants against VRE could be generated. Although bacteria are more likely to develop resistance against bacteriostatic drugs [40], no change in MIC for ebselen was observed in a multi-step resistance selection experiment. This is similar to a previous report where resistant mutants to ebselen could not be isolated for other Gram-positive bacteria, including *S*. *aureus* and *Bacillus subtilis* [41]. We suspect the inability of bacteria, such as VRE, to develop resistance to ebselen may be due to its multifaceted mechanism of action against bacteria that involves inhibition of several biochemical pathways in VRE as ebselen does in MRSA [21]. However further investigation is needed to corroborate this hypothesis. The failure to develop resistance *in vitro* is a predictive measure of low resistance development *in vivo*, although it is not a guarantee. This explains the rare cases of linezolid-resistant VRE observed in clinical blood and urine isolates from hospital patients [42].

As highlighted earlier, colonization of the GIT by VRE is an important precursor to subsequent invasion and infection. A key virulence factor that permits VRE to colonize to the GIT is the formation of biofilms. Biofilms are complex structures composed of bacteria and extracellular material that protect bacteria from the effect of antibiotics and the host immune system. Inhibition of VRE’s ability to form biofilms or disrupting adherent biofilms could potentially be advantageous to disrupting VRE from colonizing and expanding in the GIT. The ability of enterococci to colonize the GIT of mice has been previously correlated with the microorganism’s ability to form biofilm [43]. Thus, agents capable of interfering with biofilm formation would be advantageous for a drug intended to be used for decolonization of VRE. We thus investigated if ebselen could interfere with biofilm formation against VRE. Ebselen significantly inhibited VRE biofilm formation by 30%, at a concentration as low as 0.25×MIC. Furthermore, ebselen disrupted mature VRE biofilm by nearly 95% (at 16×MIC). The antibiofilm activity of ebselen against VRE is similar to ebselen’s antibiofilm activity against two other Gram-positive bacterial pathogens, *S*. *aureus* and *S*. *epidermidis* [21,44]. In addition, ebselen was previously reported to be a potent antivirulence agent against *Clostridium difficile* infection in mice through biochemical inhibition of *C*. *difficile* toxin B [45]. This could be an added advantage knowing that *C*. *difficile* coinfection is common in patients with VRE colonization and that *C*. *difficile* infection is a significant risk factor for VRE bacteremia in colonized patients [46,47].

After confirming the above *in vitro* activities, we sought to test the *in vivo* activity of ebselen in a VRE colonization reduction mouse model. Infection of ampicillin-pretreated mice resulted in colonization of the GIT of mice with VRE. Upon treatment with ebselen, the bacterial burden of VRE in fecal samples was significantly reduced after only three days of treatment. The effect extended and was more significant until the twentieth day of treatment. On day 20, ebselen treatment resulted in 4.5 log_10_-reduction (~99.99%) in VRE when compared to the initial bacterial load. Additionally, ebselen reduced about 90% of the burden of VRE in both the cecum and ileum content of infected mice. The dose of ebselen used in the study was 10 mg/kg. Interestingly, ebselen was proven to be safe for human use up to about 20 mg/kg [33,48]. This suggests a higher dose of ebselen could be investigated in a future study to determine if complete eradication of VRE from the GIT can occur.

The fact that ebselen has an established safety profile in humans will potentially shorten the development process and reduce its cost. Further studies need to be conducted to evaluate the effect of ebselen on the composition of the human gut microbiota. Also, the protective effect of ebselen against VRE recolonization and the probability of recurrence after cessation of ebselen treatment are yet to be evaluated. However, the data presented above indicate that ebselen has auspicious *in vitro* and *in vivo* activity and supports further investigation as a novel decolonizing agent to curb VRE infection in high-risk patient populations.

## Materials and methods

### Bacterial strains and chemicals

Bacterial isolates (Table 1) were obtained from the American Type Culture Collection (ATCC) and Biodefense and Emerging Infections Research Resources Repository (BEI Resources). Ebselen (Ark pharma, Arlington Heights, IL), linezolid (Chem-impex International, Wood Dale, IL), ampicillin (Peosta, IA), vancomycin hydrochloride (Gold Biotechnology, St. Louis, MO), gentamicin sulfate (Fisher Bioreagents, Fairlawn, NJ) and ramoplanin (Sigma-Aldrich, St. Louis, MO) were purchased from commercial vendors. Brain heart infusion (BHI), tryptic soya broth (TSB), tryptic soya agar (TSA) and enterococcosel broth were purchased from BD (Becton, Dickinson and Company, Cockeysville, MD) and phosphate-buffered saline (PBS) was purchased from Corning (Corning, NY).

### Broth microdilution assay

The minimum inhibitory concentration (MIC) of ebselen and control antibiotics (linezolid, ramoplanin and vancomycin) was assessed in accordance with the guidelines outlined by the Clinical and Laboratory Standards Institute (CLSI) [49]. Approximately 5 x 10^5^ CFU/mL of bacteria, in brain heart infusion broth, was incubated with serial dilutions of drugs at 37 °C for 16–20 hours. The MIC represents the lowest concentration that inhibited the growth of the bacteria by visual inspection. MIC_50_ and MIC_90_ are the lowest concentration of each agent that inhibited the visible growth of 50% or 90% of the tested isolates, respectively [50,51].

### Time-kill assay

*E*. *faecium* HM-952, about 10^6^ CFU/mL, in logarithmic growth phase was incubated with either 3×MIC or 6×MIC of ebselen, linezolid, or ramoplanin (in triplicate) at 37 °C for 24 hours. Samples left untreated served as the negative control. At the indicated time points, samples were taken from the bacterial suspensions, serially diluted in PBS, and plated on BHI agar plates to count bacterial CFU. Plates were incubated at 37 °C for at least 16 hours before enumerating colonies [52,53].

### Multi-step resistance selection of VRE against ebselen

To assess the ability of VRE to develop resistance against ebselen, *E*. *faecium* HM-952 was subcultured daily in the presence of subinhibitory concentrations of ebselen or control antibiotics (linezolid, ramoplanin, and gentamicin), using triplicate samples for each agent. At the end of each day the MIC of the tested isolate was determined, via the broth microdilution assay, to check for an increase in the MIC relative to the initial passage. A four-fold increase in the MIC, from the initial sample, was indicative of resistance formation as per previous reports [54,55].

### Anti-biofilm activity of ebselen

The ability of ebselen and control antibiotics (linezolid and ramoplanin) to inhibit VRE biofilm formation was tested, as described previously [26,56]. In brief, an overnight culture of *E*. *faecalis* NR-31972 in TSB was diluted 1:100 in fresh broth supplemented with 1% dextrose. The bacterial suspension was incubated at 37 °C with sub-MIC concentrations of all tested drugs (tested in triplicate) for 24 hours. To evaluate the biofilm density, media containing drugs and planktonic bacteria was discarded and the adherent biofilms were washed twice with sterile PBS. The biofilms were stained with 100 μL of crystal violet (0.1%) for 30 minutes. Excess crystal violet was washed out and the adherent stain was extracted using 95% ethanol for 45 minutes. The optical density (595 nm) for each treatment was measured using a microplate reader (SpectraMax i3x, Molecular Devices LLC, Sunnyvale, CA).

The ability of ebselen to disrupt established VRE biofilm was determined via the microtier dish biofilm formation assay, using the protocol described above. An overnight inoculum of *E*. *faecalis* NR-31972 was diluted 1:100 (in TSB + 1% dextrose) and were permitted to establish biofilm on a 96-well tissue-culture treated plate for 24 hours at 37 °C. Next, media was removed and drugs were added (in triplicate) and serially diluted. Biofilm was incubated with drugs for 24 hours at 37 °C. The biofilm mass was stained as described above.

### VRE colonization reduction mouse model

To evaluate the ability of ebselen and ramoplanin to decolonize VRE from the GIT of the mice, we followed the protocol proposed by Ubeda *et al* [12,15] with slight modification. Briefly, 8-week-old female C57BL/6 mice (Envigo, Indianapolis, IN) were housed in groups of five in individually ventilated cages. Mice were given access to food and water *ad libitum*. All the animal procedures were approved and done in accordance with the Purdue Animal Care and Use Committee (PACUC) and following the recommendation of the Guide for the Care and Use of Laboratory Animals of the National Institutes of Health. Ampicillin (0.5 g/l) was added to the drinking water for a week before the animals were orally infected with 10^8^ CFU/mL of *E*. *faecium* HM-952. Four days later animals were treated orally with ebselen or ramoplanin (10 mg/kg) daily for 20 days, while one group was left untreated. Bedding in the cage was changed regularly to avoid reinfection of mice. Fresh stool samples were collected from the mice on days 0, 5, 10, 15 and 20 post-infection. Mice were humanely euthanized on day 21 post-infection using CO_2_ inhalation and the cecum and ileum contents were aseptically collected. Stool samples, the cecum, and ileum were suspended in PBS, serially diluted and plated on enterococcosel agar plates (supplemented with vancomycin, 8 mg/mL) on the same day of collection to assess the bacterial burden present. Agar plates were incubated for 48 hours at 37° C before the colonies were counted.

### Statistical analysis

All statistical analysis was conducted using GraphPad Prism (version 7, GraphPad Software, La Jolla, CA). Biofilm inhibition data and data obtained from fecal samples were analyzed via two-way Analysis of Variances (ANOVA) followed by Dunnett’s pairwise comparison, while data obtained from cecum and ileum contents was analyzed using one-way ANOVA with post hoc unpaired t test.
